# Regulated exocytosis from astrocytes: a matter of vesicles?

**DOI:** 10.3389/fnins.2024.1393165

**Published:** 2024-05-10

**Authors:** Corrado Calì

**Affiliations:** ^1^Department of Neuroscience “Rita Levi Montalcini”, University of Turin, Turin, Italy; ^2^Neuroscience Institute Cavalieri Ottolenghi, Orbassano, Italy

**Keywords:** synaptic-like microvesicles, SLMVs, astrocyte, gliotransmission, exocytosis, volume electron microscopy, vEM, 3D reconstruction

## Introduction

The presence of secretory vesicles (synaptic-like microvesicles or SLMVs) in astrocytes capable of fine-tuning synaptic transmission, has been a topic of debate in the field. While some studies have suggested that astrocytes release gliotransmitters through SNARE-dependent vesicular exocytosis, other studies presented contradictory data suggesting that astrocytes release in a non-regulated manner, such as through lysosomes, or non-vesicular pathways such as channels (reviewed in Hamilton and Attwell, 2010; Savtchouk and Volterra, 2018). Evidence showing the presence of secretory vesicles in astrocytes *in situ* would support regulated exocytosis; nevertheless, efforts to investigate the presence of these organelles at the ultrastructural level, using electron microscopy, failed to convince skeptical scientists so far (Bezzi et al., 2004; Bergersen et al., 2012). Two important studies (Petravicz et al., 2008; Agulhon et al., 2010) heated the debate, by showing that astrocytic calcium signaling has no impact on synaptic activity (Smith, 2010). Moreover, murine models developed to impair astrocytic SLMVs exocytosis such as the dnSnare or IP3RKO (Pascual et al., 2005; Sherwood et al., 2021) show weak behavioral phenotype. Nevertheless, we still use genetic models to study astrocytic impact on synaptic activity, somehow upstreaming SLMVs exocytosis (Petrelli et al., 2020, 2023). Indeed, calcium signaling has been largely used as a proxy to study the dynamics of SLMVs release in culture (Calì et al., 2008; Cali et al., 2014; Marchaland et al., 2008; Vardjan et al., 2019; Stenovec et al., 2020; Mielnicka and Michaluk, 2021), leading to *in vivo* studies with the assumption that neuroglia functional interactions might be due to astrocytic SLMVs exocytosis (Kirchhoff, 2010; Wiedemann, 2010; Bindocci et al., 2017; de Ceglia et al., 2023). Aforementioned reasons led to a progressive abandonment of this quest, leaving the problem almost as a religious question, where believers don't need further proofs, and conversely no evidence will be enough for those who don't believe in it. Here, we review the current state of the art regarding our knowledge of exocytotic SLMVs in astrocytes.

## Ultrastructural evidence using EM

To date, the best way to study cellular ultrastructure from a morphological level, is by far Electron Microscopy (EM). In fact, by increasing resolution limit by a factor of two compared to fluorescence microscopy, it is the only technique capable of unequivocally identify nanometer-sized structures (Knott and Genoud, 2013; Boges et al., 2020). This includes small, astrocytic perisynaptic astrocytic processes (PAPs), and their organelles.

## The use of volume EM to characterize astrocytes fine morphology

Identifying processes on a single section EM micrograph could be misleading. Depending on the direction and position of the cut, a cellular process could be mistaken for something similar in its cross-section (e.g., small axons and microglial processes on a single section might have a similar round morphology and diameter). Volume Electron Microscopy (VEM) allows navigating along z-stacks, and observing single processes at multiple heights to make sure about who-is-what (Titze and Genoud, 2016). VEM became more and more common since a seminal paper was published in 2004 (Denk and Horstmann, 2004). Taking on a concept from 1981 (Leighton, 1981), it has been shown how SEM combined with an ultramicrotome and a high-resolution back scattered detector for block face imaging could be used to observe large portion of tissues with similar quality and resolution to classic serial-section TEM for biological application, and automatically cut serial sections at the same time (Denk and Horstmann, 2004). Although this technique has been originally developed to solve the so-called connectome (DeFelipe, 2010; Oh et al., 2014; Fua and Knott, 2015; Wanner et al., 2015), few papers managed to produce high-resolution 3D reconstructions of full morphologies of astrocytes by adapting VolumeEM to study glial cells (Coggan et al., 2018).

By using this approach, several labs interested in astrocytes have started an important work in the field, describing the astrocytic ultrastructure in three dimension, and quantifying parameters such as synaptic ensheathment, surface are to volume ratio (SVR), and working on ontologies to define a proper nomenclature of astrocytic processes in the parenchyma (reviewed in Calì, 2017). In the last 5 years, three of them focused on escheatment of full astrocytes on neurites, such as Calyx of Held (Heller et al., 2024), or relationships with vasculature, one of the most distinctive hallmarks of astrocytes in all regions of the CNS (Albargothy et al., 2022). Nevertheless, astrocytes participate to parenchymal homeostasis at many levels, hence many works have analyzed general aspects of structural neuro-glia relationships (Cali et al., 2019; Shapson-Coe et al., 2021; Møller et al., 2022; Turner et al., 2022; Salmon et al., 2023).

In a beautiful piece of work, authors have quantified the finest nanoarchitecture of diverse astrocytic processes, using computer vision methods specifically tailored for astrocytes (Salmon et al., 2023). To this regard, the use of VEM, combined with state-of-the-art computer vision techniques (Agus et al., 2018), could be a novel unbiased tool to identify astrocytic microdomains hosting SLMVs.

## EM sample preparation: stained or unstained?

**Figure 1:**
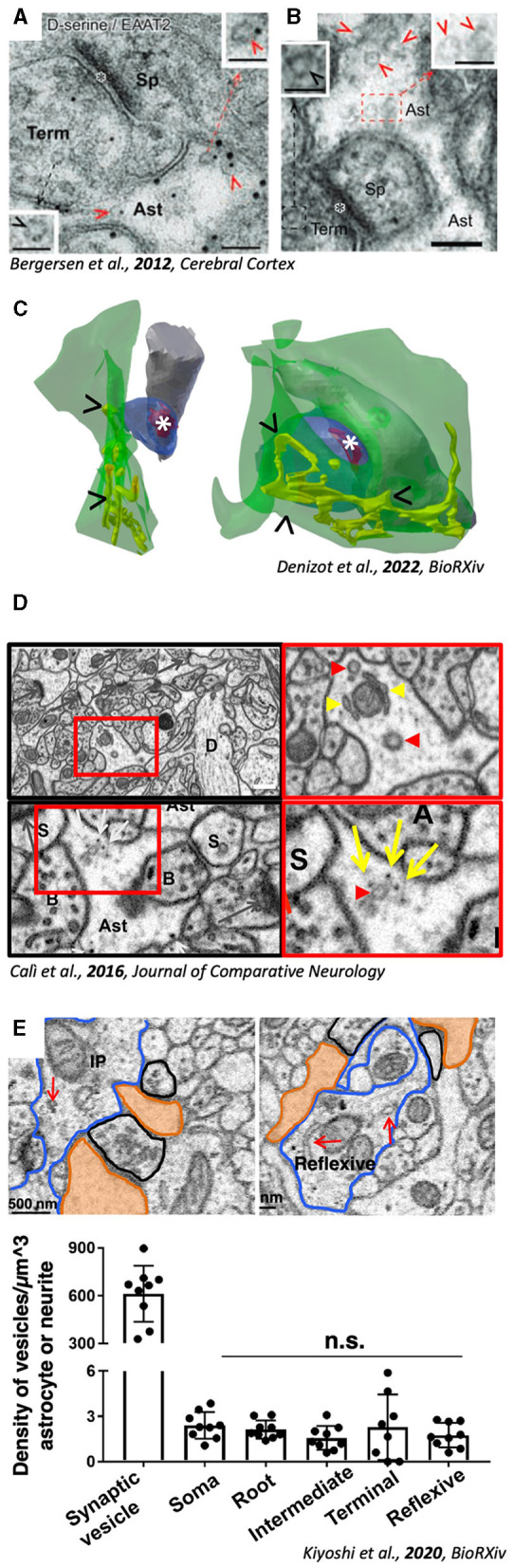
Evidence for the presence of excitatosis-competent ultrastructural compartments in PAPs. **(A)** PAP (Ast) containing SLMVs (red arrowheads). **(B)** Double Immunogold showing SLMVs (red arrows) coupled with D-Serine (small gold particles) and astrocytic membrane with EAAT2 (big gold particles). A and B, adapted with permission from Bergersen et al. (2012) **(C)** 3D reconstruction from volumeEM of PAPs (green) containing ER (yellow tubules, arrowheads “>”) juxtaposed to excitatory synapses (position of the PSD is highlighted by *). Adapted from Denizot et al. (2022). **(D)** Micrographs from hippocampal neuropil showing vesicular structures (red arrowheads) and ER tubules (yellow arrowheads) in PAPs. Adapted with Permission from Calì et al. (2016). **(E)** Micrographs showing organelles (red arrows) inside astrocytic processes. Quantification (bottom panels) reveals the significantly lower density of astrocytic vesicles compared to axonal bouton. Adapted from Kiyoshi et al. (2020).

When someone wants to approach the problem of studying astrocytes using EM, the first problem is clearly to identify them. While immunohistochemistry (IHC) allows the identification of astrocytes using specific markers [e.g., GFAP, S100Beta, glutamate transporters GLT-1 or GLAST... (Figure 1A)], it also requires a permeabilization step needed to allow antibodies to penetrate the tissue. Permeabilization physically damages membranes; while this is hardly a problem for fluorescence microscopy, it visibly degrades image quality under EM. Hence, in order to study proper ultrastructure, one should rather observe unstained tissue, with perfectly preserved membranes. Classic staining protocols allows to study brain ultrastructure (Titze and Genoud, 2016), but tissue dehydration is known to induce artifacts in particular in the preservation of extracellular matrix. Recent advancements include the use of high-pressure freezing, a technique which vitrifies tissue, using water from the sample itself fix the sample (Schertel et al., 2013; Korogod et al., 2015), supposedly preserving sample in their most natural state. Nevertheless, this technique is difficult to set up, and fails in staining large volumes of samples.

## Identification of PAPs and ultrastructural organelles in unstained tissue

Either way, identification of astrocytic processes in unstained tissue requires some training and understanding of electron micrographs. A good practice for beginners is to use empiric criteria that can be inferred from solid, well cited literature. If one has to look for astrocytes in EM micrographs then, one of the *oft* quoted sentences to this regard, is that astrocytic processes usually have an irregular shape, and a relatively clear cytoplasm (Ventura and Harris, 1999; Witcher et al., 2007; Nahirney and Tremblay, 2021). This sentence in particular comes from the classic “The fine structure of the nervous system,” from Peters et al. (1976) and possibly led to the misbelief that astrocytes are rather devoid of intracellular organelles, including ER, cisterns, or vesicles, especially in PAPs in close apposition to synapses.

## Ultrastructural basis of Ca^2+^ signaling to show rich intracellular content in PAPs

As previously mentioned, the study of astrocytic Ca^2+^ waves have been used as a proxy to characterize SLMVs dynamics, which are hard to study *in vivo*. In fact, a rich body of literature, in the first decade of 2000, has studied SLMVs exocytosis *in vitro* using astrocytic cell cultures (Calì et al., 2008, 2009; Cali et al., 2014; Marchaland et al., 2008). But, because the reliability of cultured astrocytes as a model has been challenged, supposedly because of lacking of growth factors and vascular cells during their development in classical cell culture protocols (Foo et al., 2011), findings in these papers has not been considered representative of *in vivo* physiology (Hamilton and Attwell, 2010; Verkhratsky et al., 2016; Zorec et al., 2016; Savtchouk and Volterra, 2018). The fact that it was hard to replicate studies identifying SLMVs *in vivo*, together with the misconception that astrocytes lack intracellular machinery because their cytosol is clear, convinced that also Ca^2+^ machinery was absent, in contrast to *in vitro* evidence (Marchaland et al., 2008). According to this view, a recent series of elegant studies from the Semyanov lab conducted rigorous quantifications of different types of hippocampal PAPs, showing in particular how those in close apposition to synapses are devoid of Ca^2+^ stores (Patrushev et al., 2013). Those have been found to have a high SVR, called “leaflets,” in contrast to “branches” and “branchlets,” which on the contrary contains ER (Gavrilov et al., 2018). Interestingly, a more recent paper reported and quantified the presence of organelles (ER and vesicular structures), potentially supporting Ca^2+^ signaling and synaptic tuning, in leaflets (Aboufares El Alaoui et al., 2020). Although this latter study was performed in the cortex, it supports the finding in a recent work analyzing the presence of ER in PAPs from an existing VEM dataset (Calì et al., 2016) (Figure 1C, arrowheads) to simulate calcium dynamics (Denizot et al., 2021, 2022). Simulations support the presence of ER and SLMVs in PAPs (Manninen et al., 2018, 2023; Linne et al., 2022). At present, the body of evidence of astrocytic Ca^2+^ stores *in vivo* cannot be ignored, and might help reconsider the presence of SLMVs, being Ca^2+^ their upstream triggering molecule (Calì et al., 2009; Bohmbach et al., 2018; Vardjan et al., 2019; Mielnicka and Michaluk, 2021). Moreover, in the dataset from (Calì et al., 2016), it is also possible to identify vesicular structures resembling SLMVs in size and shape (Figure 1D, red arrowheads).

## Evidence for SLMVs in astrocytes

SLMVs are round, clear, with a diameter of 50 nm, and resemble glutamatergic vesicles in excitatory boutons (Bezzi et al., 2004). Exocytotic SLMVs have been first hypothesized in the 1998 Nature paper from the group of A. Volterra (Bezzi et al., 1998), demonstrating that glutamate was released from astrocytes in a calcium-dependent manner. SLMVs were shown first in 2004 by the same group, using EM immunogold (Bezzi et al., 2004). SLMVs express VGLUTs and SNARE proteins (such as VAMPs), making them competent for regulated exocytosis. Although exocytosis from astrocytes have been extensively studied and characterized *in vitro* (Savtchouk and Volterra, 2018), the lack of reproducibility of similar data from other lab, heated the debate that created a hiatus in the field.

## State of the art on the quest of exocytotic organelles

In the last 10 years, only a few research papers have specifically characterized exocytosis of synaptic-like microvesicles from astrocytes (Cali et al., 2014; Stenovec et al., 2016, 2018, 2020; Lasič et al., 2017; Sobieski et al., 2017; Wolfes et al., 2017; Chowdhury et al., 2018; Eersapah et al., 2019; Rituper et al., 2022), all from *in vitro* models. None of these works has shown any EM micrograph, and only two are showing some fluorescence immunohistochemistry with markers for vesicular markers *in situ* (Plá et al., 2017; Di Marco Vieira et al., 2020). Looking at *in vivo* studies, two recent papers characterizing neuro-glia interplay at synaptic level, suggest the involvement of SLMVs exocytosis in the process. In Abreu et al., hippocampal plasticity is shown to be dysfunctional in dnSNARE mice lacking astrocytic d-Serine signaling (Abreu et al., 2023). De Ceglia et al. identified a subpopulation of exocytotic astrocytes in the hippocampus, expressing VLGUTs, the vesicular glutamate transporter, supposedly expressed on SLMVs (de Ceglia et al., 2023). No matter how strong the evidence or solid the work, one must admit that no novel ultrastructural evidence regarding the presence or nature of SLMVs came out in the last decade of research. The last paper on this topic was published in 2012 (Bergersen et al., 2012), using a similar approach, from the same group in Oslo (Norway) whose EM school was able to identify SLMVs in the first place (Bezzi et al., 2004) (Figures 1A, B). An important criticism to the work, is the fact that all evidence is coming from the hippocampus, leading to speculations that SLMVs exocytosis might be a feature of a subgroup of astrocytes in this specific region. Interestingly, previous cited work of PAPs and others (Gavrilov et al., 2018; Denizot et al., 2022) have also analyzed hippocampal neuropil (Figure 1).

## Large-scale VEM to look for SLMVs

One very elegant study (Kiyoshi et al., 2020; Aten et al., 2022) provided ultrastructural analysis, together with 3D reconstructions, showing organelles populating microdomains (Figure 1E). Although 3D reconstructions were not provided, single micrographs showing the organelles reveal their presence, in close proximity to synaptic structures. Interestingly, data from this paper are also coming from the hippocampus, raising again questions regarding the possible specificity of the presence of organelles only in this region only, where astrocytes are highly diverse (Viana et al., 2023). Compared to cell cultures, the presence of SLMVs in astrocytic PAPs is sparse, unlike axonal terminals (Figure 1E, bottom right panel). Distribution of SLMVs follows rules that we cannot decode just yet, which makes it difficult to identify astrocytic exocytotic subdomains. In fact, the possibility to find these by chance might be rather low, considering that a recent report shows that secretory astrocytes accounts for only 8% of all astroglial cells in the ventral hippocampus, and 24% in the dorsal hippocampus (de Ceglia et al., 2023).

## Conclusions

The presence of secretory synaptic-like microvesicles in astrocytes has a solid base of correlative data, suggesting that astrocytes participate to neuronal signaling by fine tuning synaptic transmission in a fast and highly-regulated manner. Nevertheless, ultimate proof, to show them under electron microscopy in independent studies from different labs, is still missing. Few recent studies (Aboufares El Alaoui et al., 2020; Aten et al., 2022; Denizot et al., 2022) might play an important role in re-opening a game that seemed closed a decade ago, by taking advantage of VEM. Moreover, an increasing number of labs acquiring high-resolution, large volumes, under EM, are sharing data with a larger community, a wonderful practice allowing the re-use of precious dataset for other purposes; this could be key to extend this quest to labs that are not directly working using electron microscopy.

Questions that might help to find an answer are:

- Do exocytotic microdomains in astrocytes exists?
- Which types of synapses benefit from gliotransmission?
- Are astrocytic SLMVs clustered like in synaptic boutons?
- Are SLMVs an exclusive feature of hippocampal synapses?

In the end, those who don't believe we went to the moon often cite the lack of photos of the landing sites, and that we can't really see any equipment left there using our imaging systems. This does not really mean that Neil Amstrong did not put his foot on the moon in 1969; simply, we are still not that good at looking into it.

## Author contributions

CC: Conceptualization, Funding acquisition, Visualization, Writing – original draft, Writing – review & editing.
